# Supplementary material for: Identifying multi-resolution clusters of diseases in ten million patients with multimorbidity in primary care in England
Source: Commun Med (Lond). 2024 May 29;4:102. doi: 10.1038/s43856-024-00529-4 (PMC11137021; doi:10.1038/s43856-024-00529-4)
Supplement: Supplementary file 2 — Supplementary Information [file 43856_2024_529_MOESM2_ESM.pdf]

Supplementary Information

Identifying multi-resolution clusters of diseases in ten million patients with multimorbidity in primary care in England

Thomas Beaney[1,2], Jonathan Clarke[2], David Salman[1,3], Thomas Woodcock[1], Azeem Majeed[1], Paul Aylin[1], Mauricio Barahona[2]

1. Department of Primary Care and Public Health, Imperial College London, London, W6 8RP, United Kingdom
2. Centre for Mathematics of Precision Healthcare, Department of Mathematics, Imperial College London, London, SW7 2AZ, United Kingdom
3. MSk Lab, Department of Surgery and Cancer, Faculty of Medicine, Imperial College London, London, UK

Corresponding Author:
Dr Thomas Beaney
Department of Primary Care and Public Health, Imperial College London, London, W6 8RP, United Kingdom
Email: thomas.beaney@imperial.ac.uk

Supplementary Methods

Diagnostic codes were mapped to diseases using code lists developed for the CALIBER study, which have been adapted for use in multimorbidity and CPRD Aurum.[43,44] We reviewed the codes in these lists, and made amendments to the code lists for diabetes, to remove Type 1 and Type 2 codes from the other/unspecified code list. We added chronic primary pain to the set of included conditions and created a new code list. Previous studies of multimorbidity in primary care settings have found a high prevalence and burden of chronic pain.[45,46]

We created a list of 253 known disease association pairs. These were put together by three co-authors with clinical experience, TB, JC and DS. Each of the 212 LTCs were considered for inclusion, and known associations were created using the British Medical Journal Best Practice guidelines as a source, combined with clinical interpretation. An initial set of associations was developed by TB and each code pair was reviewed and discussed with JC and DS and refined further. We aimed to generate a list of well-established disease associations, and excluded those with a low evidence-based, or those which were non-specific (for example, depression is often listed as a complication of LTCs).

We compared each of the GloVe, CBOW and SG models using the default parameter settings. We then altered each parameter in turn. For Word2vec models, we experimented with:

- Using vector sizes of 10 or 30. Vector dimensions ranging from 100-300 are commonly used, for vocabularies in the range of 10,000 or more. Given we were using only 212 unique diseases, suggested a vector size of 10 or 30 would be sufficient.
- Reducing the window size to 2 (from the default 5), given sequences were shorter than for many documents in natural language.
- Reducing the negative sampling windows to 2 (from the default 5) given shorter sequences.
- Reducing the down-sampling of frequent codes to 0 (from the default 0.001).

For GloVe models, we experimented with:

- Vector sizes of 10 or 30, as above.
- Reducing the window size to 2 (from the default 5), given sequences were shorter sequences.
- Learning rates of 0.01, 0.05 and 0.1.

In each case, we trialled models over 10 or 50 epochs for CBOW and SG, and steps for 10 to 150 for GloVe given that GloVe was computationally quicker to run. We ran CBOW and SG on unique (Table 3a) and multiple (Table 5a) codes and GloVe on unique (Table 4) and multiple (Table 6) codes. For the best performing CBOW and SG models, we then trialled runs over 10-50 epochs in steps of 10 to find the optimal number of epochs for unique (Table 3b) and multiple (Table 5b) codes. The best-performing models are highlighted in each table in bold.

Supplementary Table 1: Socio-demographic characteristics of the study population

Patient characteristic	Total	Percent
**Age (years)**		
Mean (SD)	52.2 (20.0)	
Median (IQR)	52 (36 – 68)	
18-29	1722749	16.3%
30-39	1492148	14.1%
40-49	1754523	16.6%
50-59	1700192	16.1%
60-69	1504689	14.2%
70-79	1265502	12.0%
80+	1139429	10.8%
**Gender**		
Female	5645522	53.4%
Indeterminate	263	<0.1%
Male	4933447	46.6%
**Ethnicity**		
White	7727978	73.0%
South Asian	684792	6.5%
Black	397900	3.8%
Other	166501	1.6%
Mixed	132468	1.3%
Missing	1469593	13.9%
**IMD* decile**		
1 (least deprived)	1107779	10.5%
2	1042599	9.9%
3	1080575	10.2%
4	1089697	10.3%
5	1007896	9.5%
6	1067110	10.1%
7	1090890	10.3%
8	1069964	10.1%
9	1053205	10.0%
10 (most deprived)	958965	9.1%
Missing	10552	0.1%
**Number of disease codes per patient**		
Unique codes		
Mean (SD)	6.51 (4.5)	
Median (IQR)	5 (3 – 9)	
Multiple codes		
Mean (SD)	29.1 (49.4)	
Median (IQR)	13 (6 – 33)	
**Total**	**10579232**	

* IMD = Index of Multiple Deprivation

Supplementary Figure 1: Boxplots of distribution of codes per patient for unique versus multiple code sequences

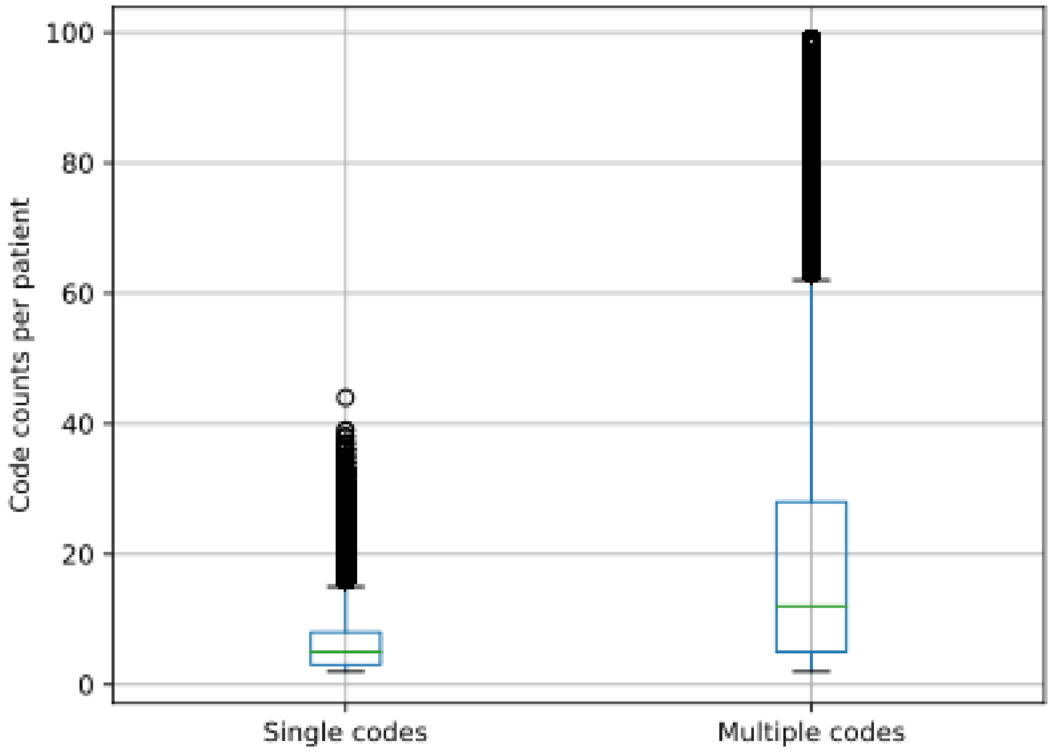

Note: patients with code sequences >100 excluded from figure to aid comparison

Supplementary Table 2: Code counts for each disease, when using unique or multiple code sequences

Disease	Count including multiple code sequences	Count including unique code sequences
Hypertension	29299147	2566996
Obesity	26738855	3889266
Raised Total Cholesterol	21254448	5408007
Diabetes Mellitus: other or not specified	17930916	1088793
Depression	14764407	2578030
Type 2 Diabetes Mellitus	12905910	949518
Asthma	12455656	2040019
Raised LDL-C	11582571	4040151
Anxiety disorders	11044361	2588744
Raised Triglycerides	10372773	2931317
Dermatitis	7717177	3220014
Low HDL-C	7269770	2196930
Osteoarthritis (excl spine)	6149283	1594700
Enthesopathy and synovial disorder	5720143	2676007
Thyroid Disease	5321262	751946
COPD	4581144	396214
Allergic and chronic rhinitis	4492882	2128435
Coronary Heart Disease (not otherwise specified)	4172594	522956
Acne	3546267	1482915
Atrial Fibrillation	3428365	477351
Vitamin B12 deficiency anaemia	2841474	360322
Migraine	2836992	1124186
Gastro-oesophageal reflux disease	2659603	1186284
Substance Misuse	2460092	314043
Chronic Kidney Disease	2380427	703739
Epilepsy	2274387	232209
Hearing loss	2225559	1230130
Abdominal Hernia	2181087	960660
Alcohol Misuse	2162303	905096
Psoriasis	2079122	527783
Stable Angina	2048244	418691
Irritable bowel syndrome	1987218	891587
Cataract	1828115	819481
Gout	1819622	446715

Dementia	1731948	308173
Rheumatoid Arthritis	1720518	119212
Osteoporosis	1534641	422911
Chronic sinusitis	1391537	774486
Heart failure	1355406	262517
Diabetic Eye Disease	1352166	412640
Diverticular Disease	1330943	546711
Gastritis and duodenitis	1284759	755216
Spondylosis	1283953	567301
Urinary Incontinence	1257820	569417
Anaemia: other	1257406	582950
Type 1 Diabetes Mellitus	1256770	79626
Stroke: not otherwise specified	1206360	284057
Diaphragmatic hernia	1196712	510601
Myocardial Infarction	1192253	274607
Schizophrenia	1148376	121606
Glaucoma	1139439	237090
Benign Prostatic Hyperplasia	1133005	317557
Primary Malignancy: Breast	1067346	196856
Chronic Fatigue Syndrome	1027070	244837
Polymyalgia Rheumatica	982877	112479
Seborrheic dermatitis	978036	593671
Intervertebral disc disorders	977271	418824
Primary Malignancy: Skin	972381	381845
Primary Malignancy: Prostate	953167	124710
Rosacea	842120	353502
Tinnitus	773209	476516
Peripheral Vascular Disease	742257	156508
Transient ischaemic attack	731052	200117
Intellectual disability	685046	93963
Dysmenorrhoea	683534	425299
Bipolar affective disorder and mania	666270	66497
Peripheral Neuropathy	647232	305229
Ulcerative colitis	639792	83516
Chronic primary pain	634565	125906
Venous thromboembolic disease (Excl PE)	590138	208586
Crohn's disease	577766	56842
Bronchiectasis	470411	78282
Parkinson's disease	457306	51106
Multiple sclerosis	435261	36334
Personality disorders	427160	111798
Primary Malignancy: Bowel	413092	88252

Macular degeneration	392186	173572
Neuropathic Bladder	381732	179425
Endometriosis	369765	149537
Fibromatosis	333800	149817
Nonrheumatic aortic valve disorders	310466	123227
Psoriatic Arthritis	307737	39529
Coeliac disease	306232	56348
Sleep apnoea	303599	159192
Obsessive-compulsive disorder	295214	73994
Spinal stenosis	283930	96146
Visual impairment and blindness	276175	165513
Raynaud's syndrome	268424	160595
Ischaemic stroke	262845	73605
Barrett's oesophagus	261318	75657
Anterior and Intermediate Uveitis	252470	108705
Hyperkinetic disorders	239081	67307
Supraventricular tachycardia	220132	71141
Pancreatitis	220127	78478
Primary Malignancy: Melanoma	217889	73642
Trigeminal neuralgia	215428	68213
Autism and Asperger's syndrome	211600	66932
Polycystic ovarian syndrome	202658	102158
Primary Malignancy: Lung	198855	46747
Nonrheumatic mitral valve disorders	193623	101391
Fatty Liver	188023	129813
Eating Disorders	185196	62384
Non-Hodgkin Lymphoma	182496	37713
Chronic viral hepatitis	180366	68204
Lupus Erythematosus	174633	23093
Folate deficiency anaemia	172387	122701
Unstable Angina	171174	81083
Cirrhosis	168159	44633
Hidradenitis suppurativa	165282	58930
Primary Malignancy: Bladder	161912	39012
Retinal vascular occlusions	158206	65224
Meniere's Disease	147148	42593
Ankylosing spondylitis	146578	24988
Leukaemia	145118	27463
Giant Cell arteritis	144602	22595
Secondary Malignancy: other	136672	44026
Primary Malignancy: other	134340	39221
Glomerulonephritis	128601	31738

Diabetic Neuropathy	125467	49135
Alcoholic liver disease	124724	39213
Obstructive and reflux uropathy	123707	73849
Sarcoidosis	122670	29707
Alopecia areata	120010	83387
Cardiomyopathy: other	115723	26421
Scleritis and episcleritis	103197	65745
Collapsed vertebra	102459	48319
Secondary Thrombocytopaenia	100387	50671
Pleural effusion	100035	60843
Benign essential tremor	99167	46059
Plasma Cell Malignancy	97358	12716
Hyperparathyroidism	96993	37320
HIV	95411	28909
Pulmonary Fibrosis	94002	24260
Vitiligo	91178	58191
Congenital Septal Defect	88317	45437
Thrombophilia	80592	47077
Primary Malignancy: Oropharyngeal	79258	14537
Subarachnoid haemorrhage	77044	28692
Cerebral Palsy	76283	17950
Intracerebral haemorrhage	73679	23640
Spondylolisthesis	72284	38292
Primary Malignancy: Ovary	71977	18471
Primary Malignancy: Oesophageal	71675	12922
Autoimmune liver disease	67481	11337
Abdominal Aortic Aneurysm	61002	27922
Sjogren's Syndrome	59763	16045
Primary Malignancy: Brain	59613	11501
Primary Malignancy: Kidney	58376	16751
Primary Malignancy: Uterus	56829	18566
Right bundle branch block combinations	56673	45121
Autonomic Neuropathy	55869	21629
Left bundle branch block	54000	35188
Hyposplenism	52991	18258
Primary Malignancy: Thyroid	50026	9331
Myasthenia gravis	48537	5926
Primary Thrombocytopaenia	48052	17594
Chronic Cystitis	44829	15198
Cystic Fibrosis	44438	3721
Dilated cardiomyopathy	43434	11035
Hodgkin Lymphoma	42511	10450

Polycythaemia vera	41335	12183
Primary Malignancy: Testis	40686	12782
Primary Malignancy: Cervix	40077	13536
Primary Malignancy: Pancreas	39440	8951
Myelodysplastic Syndrome	37362	8456
Spina bifida	37095	15910
Other haemolytic anaemias	36353	14471
Scleroderma	35985	5910
Sickle Cell Disease	34844	4637
Idiopathic Intracranial Hypertension	34066	8281
Hypertrophic Cardiomyopathy	33974	8236
Down's syndrome	33448	7578
Oesophageal varices	33382	12986
Pleural plaque	32066	18482
Secondary Malignancy: Liver	31564	15696
Atrioventricular blocks	30841	22967
Primary Malignancy: Stomach	29912	6400
Asbestosis	29396	13183
Ventricular tachycardia	28726	12501
Motor neurone disease	28165	4528
Rheumatic Valve Disorder	28126	9069
Secondary polycythaemia	24811	11254
Primary pulmonary hypertension	23353	11248
Hypersplenism	22884	15329
Aplastic anaemias	22203	9996
Endometrial hyperplasia and hypertrophy	20449	16030
Thalassaemia	20343	12424
Posterior Uveitis	18670	9540
Multiple valve disorder	18084	10842
Primary Malignancy: Liver	17852	4702
Immunodeficiencies	17075	5889
Hypertrophic Nasal Turbinates	16713	14817
Secondary Malignancy: Brain	15639	6079
Primary Malignancy: Bone	15239	3893
Pericardial Effusion	14946	9584
Secondary pulmonary hypertension	14275	7193
Primary Malignancy: Biliary Tract	14152	3471
Portal hypertension	13793	8128
Secondary Malignancy: Bone	12958	4862
Hepatic failure	11139	5237
Sick sinus syndrome	10286	5627
Secondary Malignancy: Lung	8064	3608

Angiodysplasia of colon	7116	4165
Secondary Malignancy: Lymph Nodes	6846	4075
Primary Malignancy: Mesothelioma	4267	994
Enteropathic arthropathy	3363	1125
Tubulo-interstitial nephritis	3052	1462
Secondary Malignancy: Peritoneum	1914	992
Secondary Malignancy: Adrenal Gland	274	171
Secondary Malignancy: Bowel	206	114
Secondary Malignancy: Pleura	157	54
Primary Malignancy: Multiple Sites	97	40

Supplementary Figure 2: Scree plot from MCA, for the first 30 dimensions

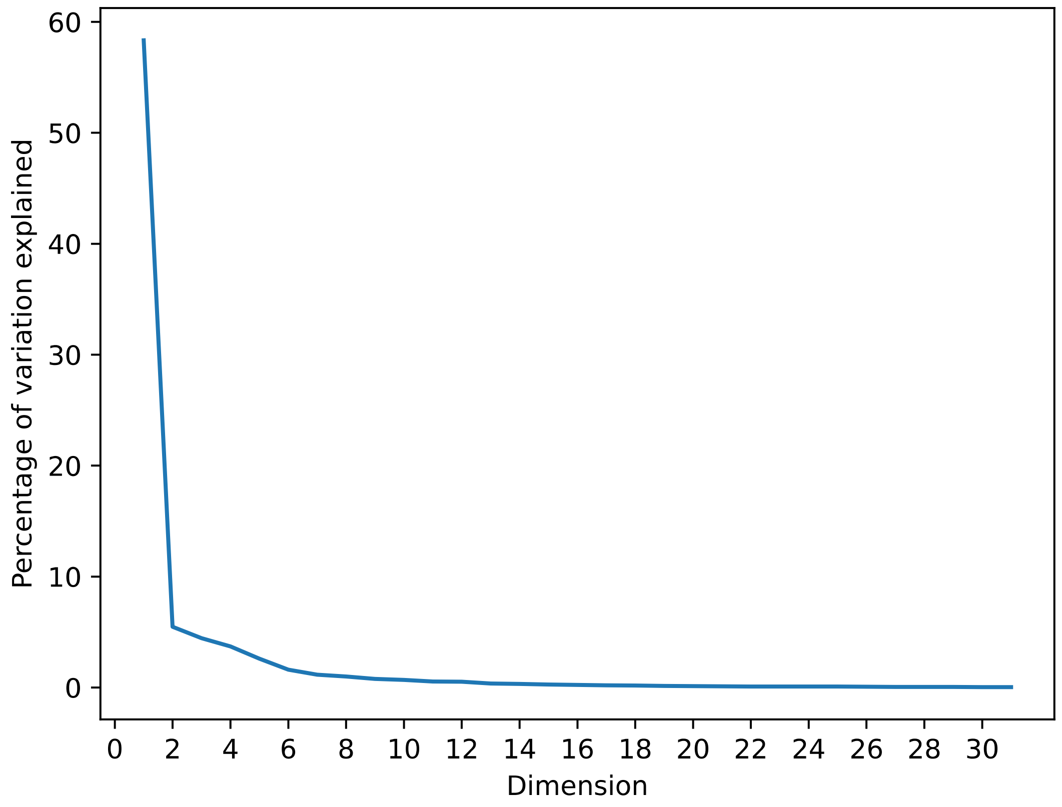

Supplementary Figure 3: Coordinates from the first two dimensions from MCA, coloured by disease system

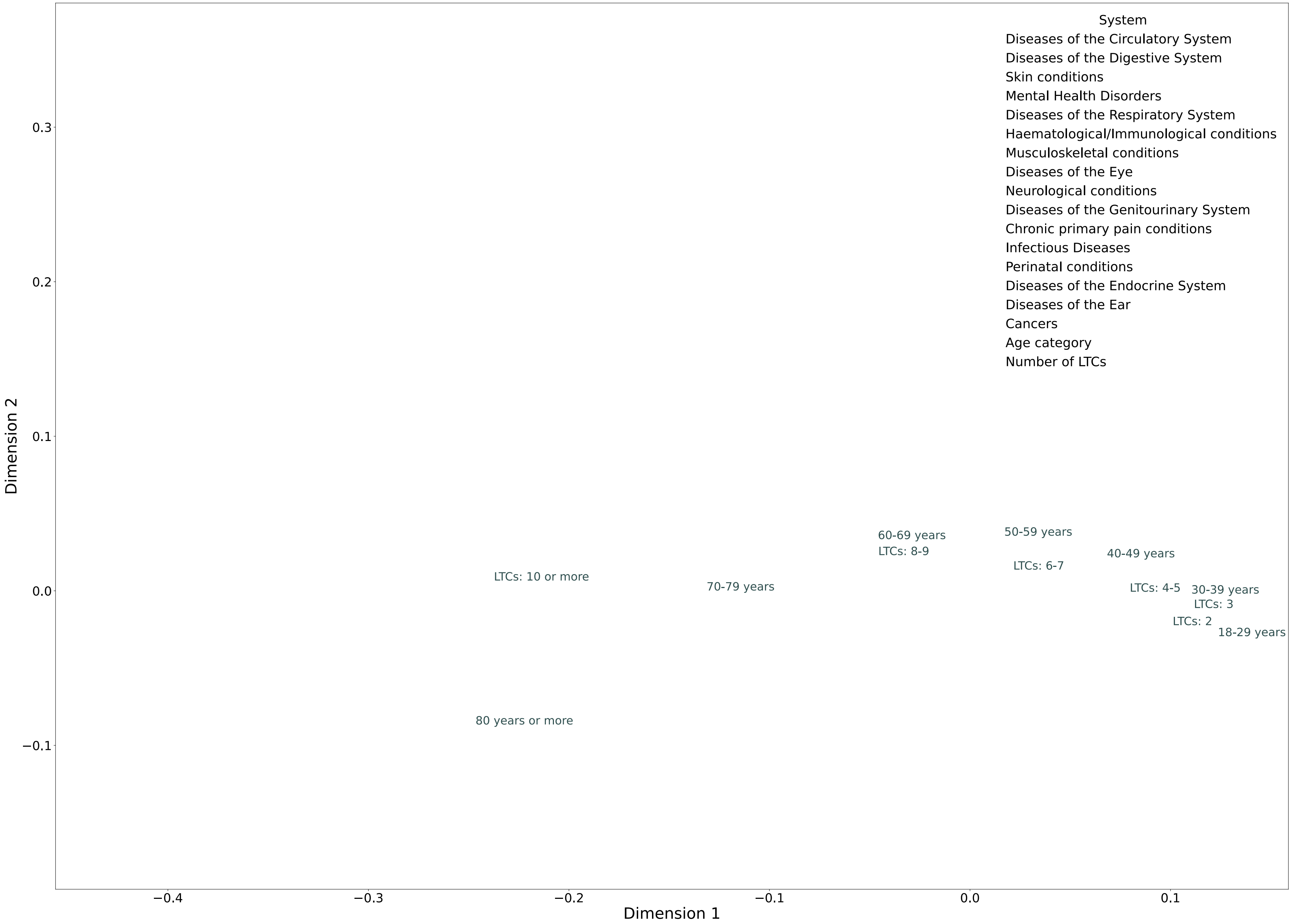

An interactive version of the figure is available at: https://tbeaney.github.io/MMclustering/mca_fig.htm

Supplementary Table 3a: Performance of Word2vec models with different hyperparameters for unique disease codes only, ordered by performance

Model	Vector size	Window	Negative	Sampling	Epochs	Top 10	Model	Vector size	Window	Negative	Sampling	Epochs	Top 10
**CBOW**	**10**	**2**	**2**	**0.001**	**10**	**42.1**	**SG**	**10**	**2**	**2**	**0.001**	**10**	**47.6**
CBOW	10	2	5	0.001	10	41.7	SG	10	5	2	0.001	10	46.2
CBOW	10	2	2	0.001	10	40.7	SG	10	2	2	0.001	10	46.0
CBOW	10	2	5	0.001	10	40.1	SG	10	5	2	0.001	10	46.0
CBOW	10	5	2	0.001	10	38.3	SG	10	2	5	0.001	10	44.9
CBOW	10	5	2	0.001	10	35.2	SG	10	5	5	0.001	10	44.3
CBOW	10	5	5	0.001	10	34.6	SG	10	5	5	0.001	10	44.1
CBOW	10	5	5	0.001	10	34.4	SG	10	2	5	0.001	10	44.1
CBOW	30	2	2	0	50	32.6	SG	30	2	2	0	50	33.0
CBOW	30	2	5	0.001	50	30.4	SG	30	2	2	0.001	50	32.6
CBOW	30	2	2	0	50	30.0	SG	30	5	2	0.001	50	32.4
CBOW	30	2	5	0	50	29.4	SG	30	2	5	0	50	32.2
CBOW	30	2	5	0.001	50	29.4	SG	30	2	2	0	50	31.8
CBOW	30	2	5	0	50	29.4	SG	30	5	2	0	50	31.6
CBOW	30	2	2	0.001	50	29.2	SG	30	5	2	0.001	50	31.6
CBOW	30	2	2	0.001	50	28.5	SG	30	2	5	0	50	31.0
CBOW	30	5	5	0.001	50	23.7	SG	10	2	5	0	10	30.8
CBOW	10	2	2	0	10	23.5	SG	30	5	2	0	50	30.6
CBOW	30	5	5	0	50	22.7	SG	30	2	2	0.001	50	30.2
CBOW	10	2	2	0	10	22.5	SG	10	2	5	0	10	30.0
CBOW	30	5	2	0.001	50	22.5	SG	10	5	5	0	10	30.0
CBOW	30	5	5	0.001	50	22.5	SG	30	5	5	0	50	30.0
CBOW	30	5	2	0	50	22.3	SG	10	2	2	0	10	29.8
CBOW	30	5	2	0	50	21.1	SG	30	2	5	0.001	50	28.9
CBOW	30	5	2	0.001	50	20.9	SG	30	5	5	0	50	28.9
CBOW	30	5	5	0	50	20.9	SG	10	2	2	0	10	28.7
CBOW	10	5	2	0	10	17.8	SG	10	5	5	0	10	28.1
CBOW	10	5	5	0	10	17.6	SG	30	2	5	0.001	50	27.9
CBOW	10	2	5	0	10	17.4	SG	10	5	2	0	10	27.3
CBOW	10	5	2	0	10	17.4	SG	30	5	5	0.001	50	27.1
CBOW	10	2	5	0	10	17.2	SG	10	5	2	0	10	26.9
CBOW	10	5	5	0	10	17.2	SG	30	5	5	0.001	50	25.5

Note: CBOW = continuous bag of words; SG = Skip-gram

Supplementary Table 3b: Run over different epochs for best performing models above

Model	Epochs				
	10	20	30	40	50
SG	**47.6**	47.0	46.2	46.4	46.0
CBOW	**42.1**	41.3	41.7	41.7	40.7

Supplementary Table 4: Performance of GloVe models with different hyperparameters for unique disease codes only, ordered by performance

Vector size	Window	Learning rate	Epochs	Top 10	Vector size	Window	Learning rate	Epochs	Top 10
**30**	**2**	**0.1**	**100**	**51.4**	30	5	0.05	30	32.2
30	2	0.1	150	50.6	30	2	0.01	100	31.4
30	2	0.1	50	50.6	30	5	0.01	150	31.2
30	5	0.1	50	50.2	10	2	0.05	100	31.0
30	2	0.1	30	49.2	30	5	0.05	20	30.0
30	5	0.1	150	48.6	30	2	0.05	10	28.9
30	5	0.1	100	48.4	10	5	0.05	50	28.5
30	2	0.1	20	47.8	30	5	0.05	10	26.9
30	5	0.1	30	47.8	30	5	0.01	100	25.3
30	2	0.05	150	46.6	10	5	0.05	20	25.1
30	2	0.05	100	46.4	10	2	0.05	50	24.7
30	5	0.1	20	45.8	10	5	0.05	30	24.5
30	2	0.1	10	45.5	10	2	0.05	30	24.3
30	5	0.05	150	44.7	30	2	0.01	50	23.5
10	2	0.1	100	43.9	10	2	0.05	20	23.3
10	2	0.1	150	43.9	10	2	0.05	10	22.7
10	5	0.1	100	43.7	10	5	0.05	10	17.6
10	5	0.1	150	43.7	30	2	0.01	30	16.6
30	5	0.05	100	42.1	30	5	0.01	50	15.8
30	5	0.1	10	41.5	10	2	0.01	150	14.4
10	2	0.1	50	41.3	30	2	0.01	20	13.6
10	5	0.1	50	40.7	30	5	0.01	30	12.8
30	2	0.05	50	40.5	30	2	0.01	10	12.1
10	5	0.1	30	37.9	10	2	0.01	100	11.7
10	2	0.1	30	37.7	30	5	0.01	20	11.1
30	2	0.05	30	37.4	10	5	0.01	150	10.9
10	2	0.1	20	37.0	30	5	0.01	10	9.9
30	5	0.05	50	37.0	10	5	0.01	100	7.9
10	5	0.1	20	36.0	10	2	0.01	50	7.5
10	2	0.05	150	35.4	10	2	0.01	30	6.5
10	5	0.05	150	35.2	10	5	0.01	50	6.5
30	2	0.05	20	33.4	10	5	0.01	30	6.3
10	2	0.1	10	33.2	10	2	0.01	10	6.1
10	5	0.1	10	33.0	10	5	0.01	20	6.1
10	5	0.05	100	32.8	10	2	0.01	20	5.9
30	2	0.01	150	32.4	10	5	0.01	10	5.1

Supplementary Table 5a: Performance of Word2vec models with different hyperparameters including recurrent codes, ordered by performance

Model	Vector size	Window	Negative	Sampling	Epochs	Top 10	Model	Vector size	Window	Negative	Sampling	Epochs	Top 10
**CBOW**	**30**	**2**	**5**	**0**	**10**	**45.1**	**SG**	**30**	**5**	**2**	**0.001**	**50**	**55.3**
CBOW	30	2	2	0.001	10	44.9	SG	30	2	5	0.001	50	55.3
CBOW	30	2	2	0	50	44.5	SG	30	2	2	0.001	10	55.3
CBOW	30	2	5	0	50	44.1	SG	30	2	5	0	10	54.3
CBOW	10	5	5	0.001	50	43.7	SG	30	5	2	0.001	10	54.3
CBOW	30	2	5	0.001	10	43.7	SG	30	2	2	0.001	50	54.3
CBOW	30	5	5	0	10	43.5	SG	30	2	2	0	10	53.6
CBOW	30	2	2	0	10	43.3	SG	30	2	5	0.001	10	52.8
CBOW	10	5	5	0.001	10	42.7	SG	30	2	2	0	50	52.8
CBOW	30	5	5	0	50	42.7	SG	30	5	5	0.001	10	52.0
CBOW	10	2	5	0.001	10	42.5	SG	30	5	2	0	10	51.8
CBOW	10	5	2	0.001	10	42.5	SG	30	5	2	0	50	51.6
CBOW	10	5	2	0.001	50	42.5	SG	30	5	5	0.001	50	51.6
CBOW	30	2	2	0.001	50	42.5	SG	30	2	5	0	50	51.4
CBOW	10	2	2	0.001	10	41.9	SG	30	5	5	0	50	48.6
CBOW	10	2	5	0	50	41.9	SG	30	5	5	0	10	48.4
CBOW	30	5	5	0.001	10	41.9	SG	10	2	2	0.001	10	47.2
CBOW	30	2	5	0.001	50	41.9	SG	10	5	2	0.001	10	47.2
CBOW	10	2	5	0.001	50	41.7	SG	10	5	5	0.001	10	47.2
CBOW	30	5	5	0.001	50	41.1	SG	10	2	5	0.001	10	46.6
CBOW	10	2	2	0.001	50	39.9	SG	10	2	5	0.001	50	46.0
CBOW	30	5	2	0.001	10	39.7	SG	10	2	2	0	50	45.5
CBOW	10	5	5	0	50	39.1	SG	10	2	2	0.001	50	45.3
CBOW	10	2	2	0	50	38.9	SG	10	5	2	0.001	50	45.3
CBOW	10	2	5	0	10	38.7	SG	10	5	5	0.001	50	44.9
CBOW	30	5	2	0.001	50	38.7	SG	10	2	2	0	10	44.7
CBOW	10	2	2	0	10	38.5	SG	10	2	5	0	10	44.3
CBOW	10	5	2	0	50	38.1	SG	10	2	5	0	50	43.9
CBOW	10	5	2	0	10	37.9	SG	10	5	2	0	10	43.5
CBOW	10	5	5	0	10	37.5	SG	10	5	2	0	50	43.1
CBOW	30	5	2	0	50	36.6	SG	10	5	5	0	10	42.3
CBOW	30	5	2	0	10	36.4	SG	10	5	5	0	50	41.1

Note: CBOW = continuous bag of words; SG = Skip-gram

Supplementary Table 5b: Run over different epochs for best performing models above

Model	Epochs				
	10	20	30	40	50
SG	54.3	**56.1**	55.5	55.3	55.3
CBOW	**45.1**	42.5	44.7	43.1	44.1

Supplementary Table 6: Performance of GloVe models with different hyperparameters including recurrent codes, ordered by performance

Vector size	Window	Learning rate	Epochs	Top 10	Vector size	Window	Learning rate	Epochs	Top 10
**30**	**5**	**0.1**	**100**	**51.4**	30	2	0.01	150	33.4
30	2	0.1	150	51.4	10	5	0.1	10	33.0
30	5	0.1	150	51.2	30	5	0.05	20	32.8
30	5	0.1	200	51.0	10	5	0.05	100	32.4
30	2	0.1	100	50.8	30	2	0.05	20	31.6
30	2	0.1	200	50.6	30	5	0.01	200	31.2
30	2	0.1	50	50.2	30	2	0.01	100	31.0
30	5	0.1	50	50.2	30	5	0.01	150	30.6
30	2	0.1	30	49.6	30	2	0.01	200	29.8
30	5	0.1	30	49.4	10	2	0.05	50	29.6
30	2	0.1	20	49.0	30	5	0.05	10	26.5
30	2	0.05	200	48.6	30	5	0.01	100	26.1
30	5	0.05	200	48.2	10	2	0.05	30	25.3
30	5	0.05	150	47.6	10	5	0.05	50	24.7
30	5	0.1	20	47.6	10	2	0.05	20	23.3
30	2	0.05	150	47.4	10	5	0.05	20	22.9
10	2	0.1	200	46.2	10	5	0.05	30	22.5
30	2	0.1	10	46.2	30	2	0.01	50	21.7
10	2	0.1	150	46.0	30	2	0.05	10	21.3
30	2	0.05	100	45.7	10	2	0.05	10	20.2
10	2	0.1	100	45.5	10	5	0.05	10	20.0
10	5	0.1	150	45.5	30	5	0.01	50	18.8
30	5	0.1	10	45.3	10	2	0.01	200	15.8
10	5	0.1	200	45.1	30	2	0.01	30	15.0
30	5	0.05	100	44.9	30	5	0.01	30	14.8
10	5	0.1	100	44.7	10	5	0.01	200	13.8
10	2	0.1	50	43.3	10	2	0.01	150	13.4
30	2	0.05	50	42.5	30	2	0.01	20	12.5
30	5	0.05	50	41.9	30	5	0.01	20	12.5
10	2	0.1	30	41.7	10	2	0.01	100	11.7
10	5	0.1	50	41.5	10	2	0.01	50	11.3
10	2	0.05	200	40.3	10	5	0.01	150	10.7
10	2	0.1	20	39.9	30	2	0.01	10	10.5
30	5	0.05	30	38.9	30	5	0.01	10	10.3
10	5	0.1	30	38.7	10	2	0.01	30	9.5
10	5	0.05	200	38.3	10	2	0.01	20	8.9
10	2	0.05	150	38.1	10	2	0.01	10	8.5
30	2	0.05	30	38.1	10	5	0.01	100	7.9
10	2	0.05	100	36.4	10	5	0.01	50	7.1
10	5	0.1	20	35.6	10	5	0.01	20	6.5
10	5	0.05	150	35.2	10	5	0.01	30	6.5
10	2	0.1	10	35.0	10	5	0.01	10	5.9

Supplementary Figure 4: Comparison performance of each embedding model using different number of nearest neighbours of 2, 5, 10 and 20

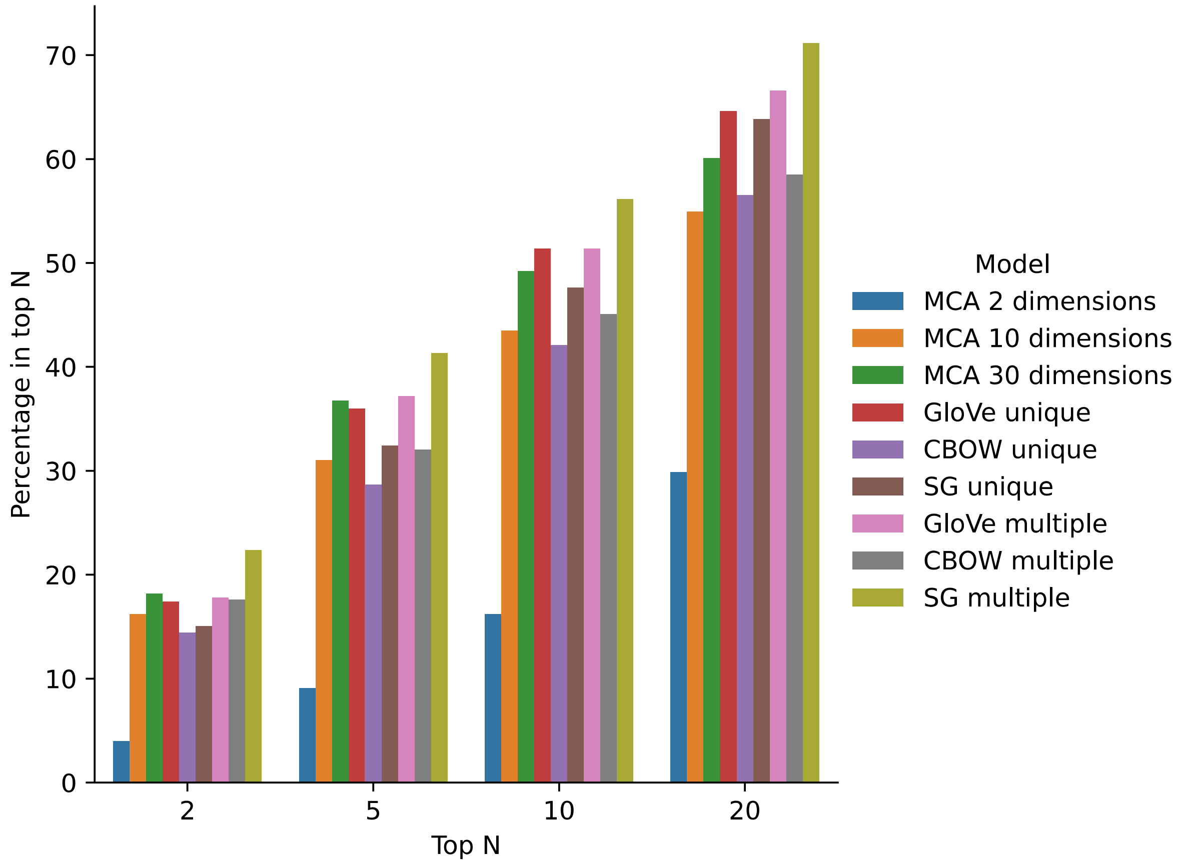

Supplementary Figure 5: Mean Jaccard similarity of the set of two diseases for each patient assigned to a disease cluster, using MCA-30 embeddings (panel A) and SG-M embeddings (panel B)

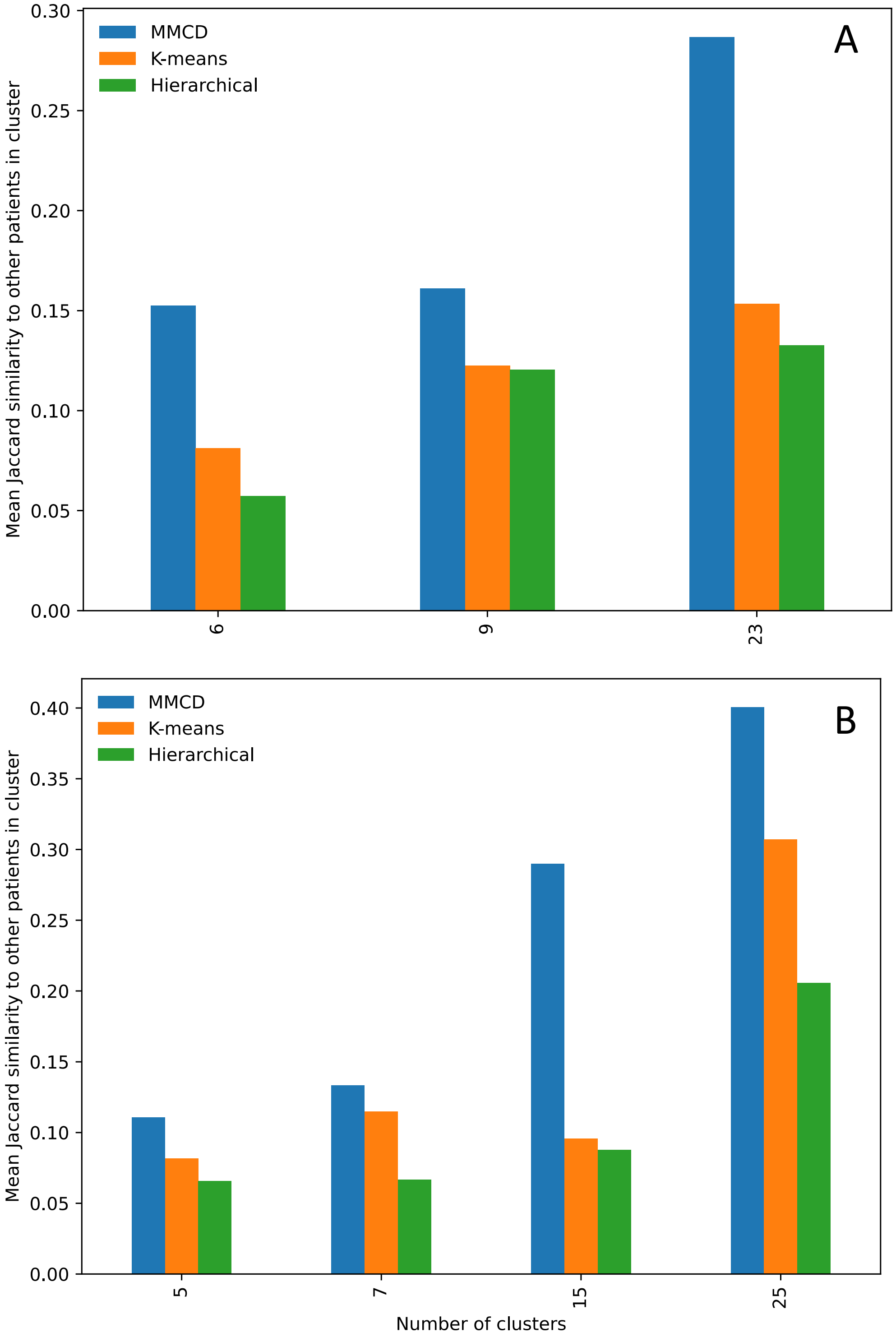

Supplementary Table 7A: Normalised variation of information (NVI) comparing assignment of diseases to clusters from MCA-30 to chapters in ICD-10

Number of clusters	NVI
23	0.60
9	0.70
6	0.75

Supplementary Table 7B: Normalised variation of information (NVI) comparing assignment of diseases to clusters from SG-M to chapters in ICD-10

Number of clusters	NVI
25	0.55
15	0.62
7	0.68
5	0.75

Supplementary Table 8: Ratio of observed to expected prevalence of co-occurrence for each pair of conditions in the thyroid and other malignancy cluster (SG-M at a 15-cluster resolution)

Disease	Primary Malignancy: Brain	Primary Malignancy: Melanoma	Primary Malignancy: Testis	Primary Malignancy: Thyroid
Primary Malignancy: Melanoma	2.29			
Primary Malignancy: Testis	1.96	1.17		
Primary Malignancy: Thyroid	1.61	2.35	0.89	
Thyroid Disease	1.61	1.36	0.45	9.29

Supplementary Table 9: Data for Figure 2 in main text

Model	Top N	Number in top 10	Percentage in top 10
MCA 2 dimensions	10	82	16.2
MCA 10 dimensions	10	220	43.5
MCA 30 dimensions	10	249	49.2
GloVe unique	10	260	51.4
CBOW unique	10	213	42.1
SG unique	10	241	47.6
GloVe multiple	10	260	51.4
CBOW multiple	10	228	45.1
SG multiple	10	284	56.1

Supplementary Table 10: Data for Figure 7 in main text (MCA embeddings, panel A)

Clustering method	Number of clusters	Odds Ratio
MMCD	23	21.0
MMCD	9	8.5
MMCD	6	6.9
K-means	23	3.4
K-means	9	3.5
K-means	6	2.2
Hierarchical	23	3.2
Hierarchical	9	3.8
Hierarchical	6	3.0

Supplementary Table 11: Data for Figure 7 in main text (SG-M embeddings, panel B)

Clustering method	Number of clusters	Odds Ratio
MMCD	25	20.5
MMCD	15	14.8
MMCD	7	12.9
MMCD	5	9.7
K-means	25	16.0
K-means	15	6.4
K-means	7	6.9
K-means	5	6.5
Hierarchical	25	16.3
Hierarchical	15	10.0
Hierarchical	7	5.8
Hierarchical	5	5.4
