# Supplementary material for: Identifying multi-resolution clusters of diseases in ten million patients with multimorbidity in primary care in England
Source: Commun Med (Lond). 2024 May 29;4:102. doi: 10.1038/s43856-024-00529-4 (PMC11137021; doi:10.1038/s43856-024-00529-4)
Supplement: Supplementary file 3 — Reporting Summary [file 43856_2024_529_MOESM3_ESM.pdf]

nature portfolio

Corresponding author(s): Thomas Beaney

Last updated by author(s): Jul 18, 2023

Reporting Summary

Nature Portfolio wishes to improve the reproducibility of the work that we publish. This form provides structure for consistency and transparency in reporting. For further information on Nature Portfolio policies, see our Editorial Policies and the Editorial Policy Checklist.

Statistics

For all statistical analyses, confirm that the following items are present in the figure legend, table legend, main text, or Methods section.

n/a	Confirmed	
☐	☒	The exact sample size (n) for each experimental group/condition, given as a discrete number and unit of measurement
☐	☒	A statement on whether measurements were taken from distinct samples or whether the same sample was measured repeatedly
☐	☒	The statistical test(s) used AND whether they are one- or two-sided<br>*Only common tests should be described solely by name; describe more complex techniques in the Methods section.*
☐	☒	A description of all covariates tested
☒	☐	A description of any assumptions or corrections, such as tests of normality and adjustment for multiple comparisons
☐	☒	A full description of the statistical parameters including central tendency (e.g. means) or other basic estimates (e.g. regression coefficient) AND variation (e.g. standard deviation) or associated estimates of uncertainty (e.g. confidence intervals)
☒	☐	For null hypothesis testing, the test statistic (e.g. F, t, r) with confidence intervals, effect sizes, degrees of freedom and P value noted<br>*Give P values as exact values whenever suitable.*
☒	☐	For Bayesian analysis, information on the choice of priors and Markov chain Monte Carlo settings
☒	☐	For hierarchical and complex designs, identification of the appropriate level for tests and full reporting of outcomes
☒	☐	Estimates of effect sizes (e.g. Cohen's d, Pearson's r), indicating how they were calculated

Our web collection on statistics for biologists contains articles on many of the points above.

Software and code

Policy information about availability of computer code

Data collection	N/A
Data analysis	We used Python version 3.8.10 and Pandas version 1.3.5 for data manipulation and management. Sankey diagrams were created in Plotly.

For manuscripts utilizing custom algorithms or software that are central to the research but not yet described in published literature, software must be made available to editors and reviewers. We strongly encourage code deposition in a community repository (e.g. GitHub). See the Nature Portfolio guidelines for submitting code & software for further information.

Data

Policy information about availability of data

All manuscripts must include a data availability statement. This statement should provide the following information, where applicable:

- Accession codes, unique identifiers, or web links for publicly available datasets
- A description of any restrictions on data availability
- For clinical datasets or third party data, please ensure that the statement adheres to our policy

This study uses patient level which is not publicly available but can be requested for users meeting certain requirements: https://cprd.com/research-applications. The code lists and embeddings generated from this work are available to download from: https://tbeaney.github.io/MMclustering/.

Human research participants

Policy information about studies involving human research participants and Sex and Gender in Research.

Reporting on sex and gender	We refer to the term 'gender', which is the variable collected in the CPRD dataset. This records 'male', 'female' and 'indeterminate'
Population characteristics	We have available, demographic information on age, sex and ethnicity. We also have linked data to markers of socioeconomic deprivation (IMD)
Recruitment	The CPRD is routinely collected data from primary care electronic healthcare record systems.
Ethics oversight	Data access to the Clinical Practice Research Datalink (CPRD) and ethical approval was granted by CPRD's Research Data Governance Process on 28th April 2022 (Protocol reference: 22_001818).

Note that full information on the approval of the study protocol must also be provided in the manuscript.

Field-specific reporting

Please select the one below that is the best fit for your research. If you are not sure, read the appropriate sections before making your selection.

☒ Life sciences ☐ Behavioural & social sciences ☐ Ecological, evolutionary & environmental sciences

For a reference copy of the document with all sections, see nature.com/documents/nr-reporting-summary-flat.pdf

Life sciences study design

All studies must disclose on these points even when the disclosure is negative.

Sample size	We used all available patients in order to maximise inferences for rare diseases, and as such, there was no sample size
Data exclusions	We included all patients aged 18 years or over, registered to a GP practice in CPRD Aurum between 1st January 2015 and 1st January 2020. Patients were censored at the earliest of date of deregistration, date of death, date of last collected data extraction from the practice, or the 1st January 2020. Patients with two or more of the diseases defined below were included.
Replication	N/A
Randomization	N/A
Blinding	N/A

Reporting for specific materials, systems and methods

We require information from authors about some types of materials, experimental systems and methods used in many studies. Here, indicate whether each material, system or method listed is relevant to your study. If you are not sure if a list item applies to your research, read the appropriate section before selecting a response.

Materials & experimental systems

n/a	Involved in the study	
☒	☐	Antibodies
☒	☐	Eukaryotic cell lines
☒	☐	Palaeontology and archaeology
☒	☐	Animals and other organisms
☒	☐	Clinical data
☒	☐	Dual use research of concern

Methods

n/a	Involved in the study	
☒	☐	ChIP-seq
☒	☐	Flow cytometry
☒	☐	MRI-based neuroimaging
